# Does Chronic Obstructive Pulmonary Disease Affect Workers' Health?

**DOI:** 10.3389/fpubh.2021.711629

**Published:** 2021-07-05

**Authors:** Li-Peng Yao, Ran Tao

**Affiliations:** ^1^Ningbo College of Health Sciences, Ningbo, China; ^2^Qingdao Municipal Center for Disease Control and Preventation, Qingdao, China

**Keywords:** COPD, workers health, probit model, SMEs, China

## Abstract

During recent decades, the elevation of workers' health has become the utmost point of concern since it is considered among the primary indicators of economies. The economies, especially the emerging ones, are primarily focusing on every indicator to uplift their businesses. Along with the other aspects, it is also required to assess the impact of Chronic Obstructive Pulmonary Disease (COPD) on workers' health conditions in small- and medium-scale enterprises (SMEs). With this aim, we are presenting a detailed analysis to reveal useful insights regarding the COPD-workers' health nexus. The sample set of 1,008 workers is working in various SMEs in Beijing and Tianjin from September, 2020. The findings infer that a rise in COPD concerning wages will uplift the worker health problems due to COPD affecting worker health. Whereas, the working condition and tools, smoking years, and health safety training have a statistically adverse effect on workers' health concerning wages. The outcomes in terms of insights would be useful for planning future perspectives.

## Introduction

Chronic obstructive pulmonary disease (COPD) has a high economic burden for each economy. Soriano et al. (1) reported that COPD becomes the third leading source of death and the sixth source of disability worldwide at the end of 2030. Therefore, COPD outcomes in a high economic burden consist of two costs; direct healthcare expenditure and indirect costs of lost productivity (2). As Woo et al. (3) covered a systematic review of six economies in Asia-Pacific named China, South Korea, Taiwan, Japan, Thailand, and Singapore. They noted that the COPD prevalence rate in Asia-Pacific is 6.2%. Whereas, at the estimates of regional level, prevalence rates ranged from a high of 13.6% in China, 8.2% in South Korea, 7% in Japan, 6.1% in Taiwan, and 5.9% in Singapore to a low of 5.3% in Thailand (4–7). The economic burden of direct costs and indirect costs of each country have the difference, showing that the government offset the high indirect costs *via* subsidy.

COPD is a medical disease that includes a group of chronic, progressive, and debilitating respiratory conditions, including chronic bronchitis and emphysema. It is the foremost cause of mortality and morbidity globally. It is observed that COPD is the fourth foremost reason for global mortality WHO (8), and its occurrence is anticipated to increase (9). COPD is considered by persistent, progressive airflow limitation and often appeared with a cough and enlarged sputum production. The smoke from industrial toxins and biomass fuels is also problematic risk factors (10). The symptoms of COPD source noteworthy damage to workers' health and quality of life, including breathlessness, physical limitations, and anxiety, resulting in the workers' efficiency in the industry. COPD signifies severe morbidity, and tobacco smoking is the leading source of COPD, and each sector of the industry is affected, especially small- and medium-sized enterprises (SMEs) have also faced problems in various economies.

No doubt, SMEs are considered the backbone of every economy, and the adverse situation of workers' health in SMEs is more alarming. Kawahara et al. (11) reported the information, risk perception, and self-efficacy factors that positively influenced the workers' health in SMEs. Working in SMEs is particularly riskier. Theoretical and empirical literature renowned that the risk of workers' health in SMEs is higher than larger enterprises (12, 13). The literature shows several common factors that can explain the performance of SMEs, and, in particular, of SMEs compared with larger enterprises, for instance, lack of financial resources, absence of workers' representation, and weak commitment of managers (14–16). The fact is that workers' health is one of the main factors of firm performance, and mostly SMEs have issues in workers' health, and COPD significantly disturbs the workers' heath in the industry.

Often, SMEs have more experience of difficulties in workers' health due to scarcer resources and are often less aware than large enterprises of costs experienced due to non-compliance (17). As Economic Census of Japan (2012) reported that 70% of workers are employed in SMEs in Japan, among these workers, larger proportions of health problems have been found in SMEs than those engaged in larger enterprises. A bulk of workers are employed by SMEs (18), which have greater work-related injury and disease rates than larger enterprises (19). SMEs tend to focus on traditional working styles normally and faced hazards (e.g., repetitive motion and chemical exposure and COPD). Lamm (20) and Antonsson et al. (21) observed that SME enterprises have limited resources, are more financially pressured, and have few work-related safety and health programs compared with their large enterprises (22). Owners of SMEs often manage manifold responsibilities, but limited resources and lack of formal training and experience could not focus on health workers (14, 20). Therefore, workers' productivity in SMEs is relatively small compared with large enterprises and difference due to COPD.

A bulk of studies observed that, as Sin et al. (23) suggest, COPD is one of the rapidly growing public health problems globally, although direct costs of COPD are more for the economy, and its severity affected workforce participation. Sullivan et al. (24) assess the economic burden of COPD and estimated the $18 billion annual direct costs of COPD in the USA, although COPD also severely affects the working-age population. COPD has the potential to delay a person's ability to work, leading to lost wages for workers, employers' working hours, and productivity losses. Some empirical studies reported the impact of COPD on workers in the agricultural sector (25–28). A few studies are conducted for worker health at the industrial sector level, for instance, Thompson et al. (29) and Gul et al. (30). Ulvestad et al. (31) reported COPD factors in tunnel workers but could not find the impact of COPD on workers' health. Lim et al. (6) noted the impact of COPD disease in households in the Asia-Pacific region and reported the importance of education in COPD management. While some empirical studies indicate that COPD disparities, trends, and their impacts on workers' health are important among SMEs, it is unclear from the literature how SME owners can manage COPD. Previous literature concludes that COPD has adversely affected workers' health in industrial and agricultural sectors. As for the previous empirical literature, the adverse impact of COPD on workers' health and productivity is not well-known.

However, no empirical research work is conducted on the impact of COPD on workers' health in SMEs in emerging economies. Therefore, this empirical study aimed to examine the concentrations of COPD and its types in the industrial samples (small and medium) of the workers in emerging one's economy and measure its hazardous effects on workers' health. The available health literature on the occupational causes of COPD is vast, but the impact of COPD on workers' health is too limited. However, no study to date has scrutinized COPD impact of workers' health nexus in SMEs to our knowledge. This study has also attempted to explore the determinants of COPD in SMEs. The remaining study is prepared as follows: the next section summarizes the previous literature, and the fourth section presents the methods and econometric analysis. Conclusions and implications are also mentioned in the final section.

## Literature Review

It is considered that COPD is one of the leading sources of mortality and morbidity among the adult population worldwide (2). They noted in developed nations that COPD is the most common reason for death after malignancies, cardiovascular diseases, and deaths related to accidents. Also, the ratio of COPD-related deaths in the total deaths is increasing day by day, with the forecast that by 2020, COPD will be the third most common source of death in the world. The highest occurrence was also noted in Africa and the lowest in Germany. COPD-related mortality in Europe varies from 10/100,000 in France, Greece, Spain, and the UK, up to around 40/100,000 in Belgium, Germany, Denmark, Norway, Sweden, Russia, Hungary, and Romania. In Poland, symptoms of chronic damage of airflow have also existed in 4.9% of females and 8.5% of males. The numbers of death vary in each region of the world. Smoking is observed as one of the strongest links with a relative risk of 2.3 in females and 4.3 in males.

A study by Pływaczewski et al. (32) noted that in the high age group between 41 and 72, COPD was more detected in 10.3% of females and 10.9% of males, while the relative share of males is more compared with females. Among smokers, respondents above the age of 40 participate in testing, and 23% had COPD. In 2001, patients related to COPD were 24.2/10,000 for females and 41.9/10,000 for males, while males faced more problems than females. Similarly, in 1999–2000, COPD was the main cause of death in 1.3% of females and 2.6% of males. In the world, the risk factors of COPD in the male are 4.3% and 2.3% in female, as observed by Szczyrek et al. (28).

Air pollution in the home environment is less significant but yet very important. In developing economies, exposure to home-based air pollution, initiated by the use of biomass as fuel for heating and cooking, contributes to the whole COPD morbidity. The burning of biomass is more responsible for COPD as compared with outdoor pollution or smoking. A high occurrence of COPD among non-smoking females in Africa, the Middle East, Asia, and America is considered to be caused by the extensive usage of cooking fuels for biomass (13, 33). Clean air in the working place is also very important especially for work efficiency. Eduard et al. (26) observed that the COPD factors are strongly linked with chronic bronchitis and exposure to hydrogen sulfide, ammonia, and inorganic dust. Research-related agricultural workers (25) considered the prevalence of bronchitis in French dairy farmers and decided that they are at high risk of bronchial obstruction and chronic bronchitis. Similarly, Greskevitch et al. (34) examined the proportional mortality ratios (PMRs) for respiratory conditions among six agricultural sectors, such as crop farmworkers, farm managers, livestock farm workers, forestry, and fishery and landscape and horticultural workers. They also examined prevalence ratios (PRs) for 12 respiratory conditions among two agricultural sectors: agricultural workers and farm managers. Data analysis also revealed that livestock farmworkers and crop farmworkers had significantly raised mortality linked with respiratory conditions. Mortality for hypersensitivity pneumonitis was higher 10–50 times than expected. Whereas, horticultural and landscape workers had also significantly raised mortality for mediastinum and lung abscesses and chronic airway obstruction. Similarly, forestry workers had higher mortality than expected for pneumonia, TB, and chronic airway obstruction. The occurrence of asthma was also higher among smoker's workers.

Likewise, Monsó et al. (27) also found a strong relationship between COPD, endotoxin exposure, and dust. Similarly, Eduard et al. (26) highlighted the issues of agricultural workers and compared the possibility of COPD among livestock farmers and crop farmers. Crop farmers are less likely to suffer from COPD. They also measured the subjects' lung function and their effect of exposure to living agents. Exposure to most of the living agents expected respiratory disease and found a significant impact on human life. Omland et al. (35) found a strong, consistent, and primarily significant link between COPD and occupational exposures across and within industry groups, and they also found risk factors for COPD, such as vapors, dust, gas, and fumes. Lim et al. (6) surveyed the EPIC Asia region and concluded that a high prevalence of COPD in the contributing Asia-Pacific regions shows a substantial social and economic burden of the COPD disease in this region. This study also suggests that patient and physician education can also enhance and improve the health efficiency of employed workers in the region. Similarly, Gershon et al. (36) reported that social and economical are the most influential factors of workers' health. They reported the significant inverse links between socioeconomic and COPD outcomes. Nurmagambetov et al. (37) reported the key sources of mortality and morbidity in the USA. In 2000, they noted that 10.5 million people had faced the problem of COPD, while 7.2 million employed workers were below 65 years of age. While the cost of COPD to workers is too high, the cost could be reduced by providing more outpatient facilities to COPD patients. Kawahara et al. (11) noted that risk perception, self-efficacy, and information regarding COPD are improved in the SMEs' daily-based productivity. Thus, socioeconomic factors are a key source of COPD and also adversely affect the workers' health.

## Data Collection and Computational Strategy

Beijing, an industrial power, is taken as a case study to explore the impact of COPD on workers' health. It has a significant industrial size and power, and sister city Tianjin, a hub of aerospace, automobile, mobile/cellphones, and energy-related product. The sample set of 1,008 workers is working in various SMEs in Beijing and Tianjin from September, 2020. A closed-ended, Likert 5 scaling, the questionnaire has been distributed online by utilizing the survey monkey platform (https://www.surveymonkey.com/). The questionnaire consists of a set of attributes of workers, i.e., demographic attributes, impact variables, and target binary variable, i.e., a health worker.

Computational tools such as MS Excel, SPSS, and Stata have been utilized to conduct the empirical analysis. The sample set was collected from the target group, SME workers, and requested to accurately fill the demographic attributes, impact variables, and target variables. For this, a bilingual questionnaire, English and Chinese, has been developed to understand SME workers. Accordingly, they can provide a reliable dataset to conduct empirical analysis. Logistic regression, correlation, and frequency distribution analysis have been conducted to explore the linkages among COPD and workers' health, respectively.

## Model Description

In light of the maximum likelihood estimation approach, this study uses a binary logit regression approach to reveal the nexus of COPD and workers' health. The considered model elaborates whether or not COPD affects the workers' health and pinpoints features of SMEs workers' health. The logistic regression can be stated as:

(1.1) $$\begin{array}{l} \text{Prob }\left(\text{worker}{\text{s}}^{\text{′}}\text{ health}=\text{WH }=1\right)\;=\;\frac{e^{\text{z}}}{1+e^{\text{z}}} \end{array}$$

where *e* = natural logarithm base

(1.2) $$\begin{array}{l} \text{WH}=\text{Does the COPD affect the WH}\\ \;=\left\{\begin{array}{c} \text{WH}=1\text{ if COPD affect the WH }\\ \text{WH}=0\text{ if COPD does not affect the WH} \end{array}\right\}\; \end{array}$$

In light of the introduction and literature review sections, the present study contributes to estimating the nexus of COPD and workers' health empirically, and its functional form is written as follows:

(1.3) $$\begin{array}{l} \text{WH }=\;f\;\left(\text{COPD},\text{ WCT},\text{SK},\text{ WG},\text{HS}\right) \end{array}$$

(1.4) $$\begin{array}{l} \text{WH }=\;{\text{γ}}_{0}+{\text{γ}}_{1}\text{COPD}+\;{\text{γ}}_{2}\text{WCT}+\;{\text{γ}}_{3}\text{SK}+{\text{γ}}_{4}\text{ WG}\\ \;+{\text{γ}}_{5}\mathrm{HS}+\mu_{\text{i}} \end{array}$$

In **Equation 1.4**, WH is workers' health (target variable), whereas COPD is chronic obstructive pulmonary disease and measured by respiratory symptoms, WCT is working condition and tools, SK is smoking, WG is wages, and HS is health safety training. COPD is an impact variable, while WCT, SK, WG, and HS are controlled variables in the estimated model. μ_*i*_ is an error term measuring the extent to which the model cannot fully explain future intentions.

## Data Analysis and Result Discussion

### Graphical and Correlation Analysis

**Figure 1:**
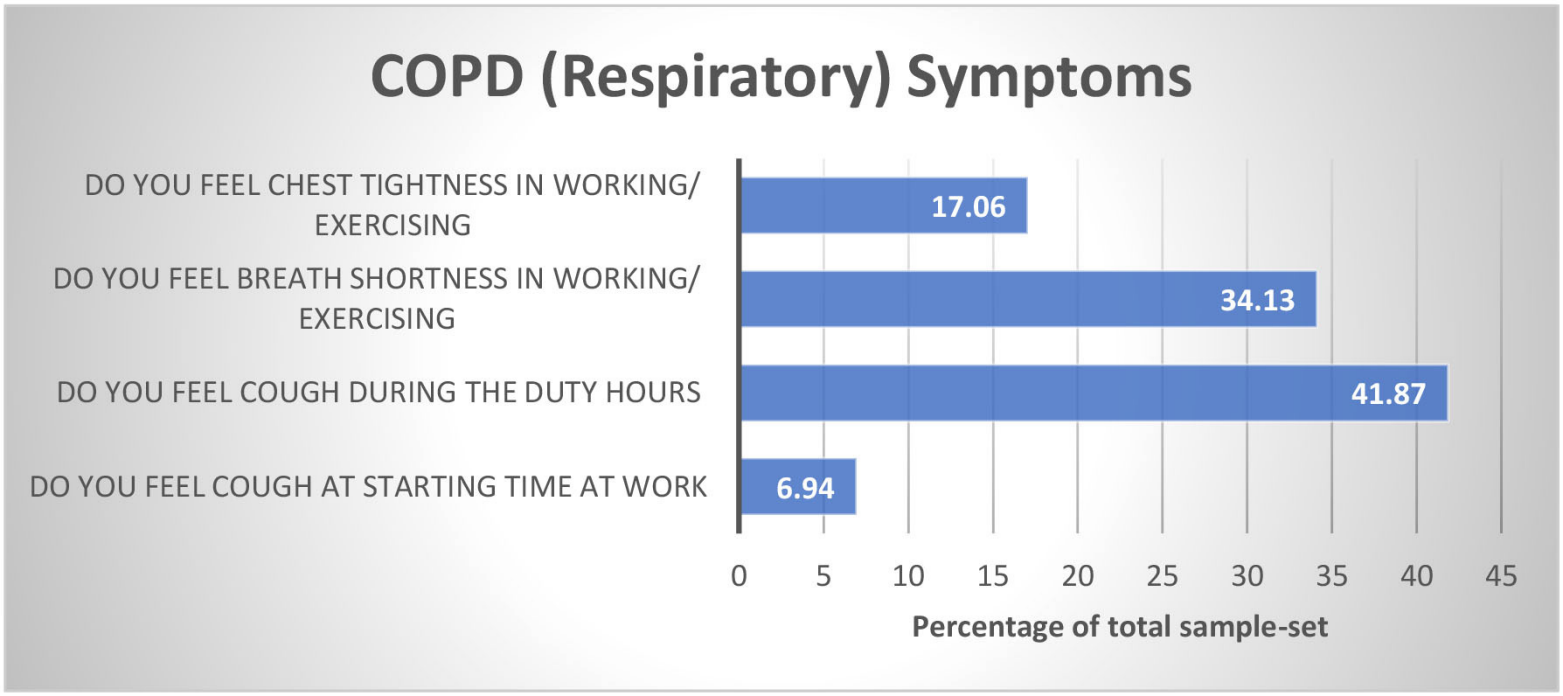
COPD symptoms in Chinese SMEs.

**Figure 2:**
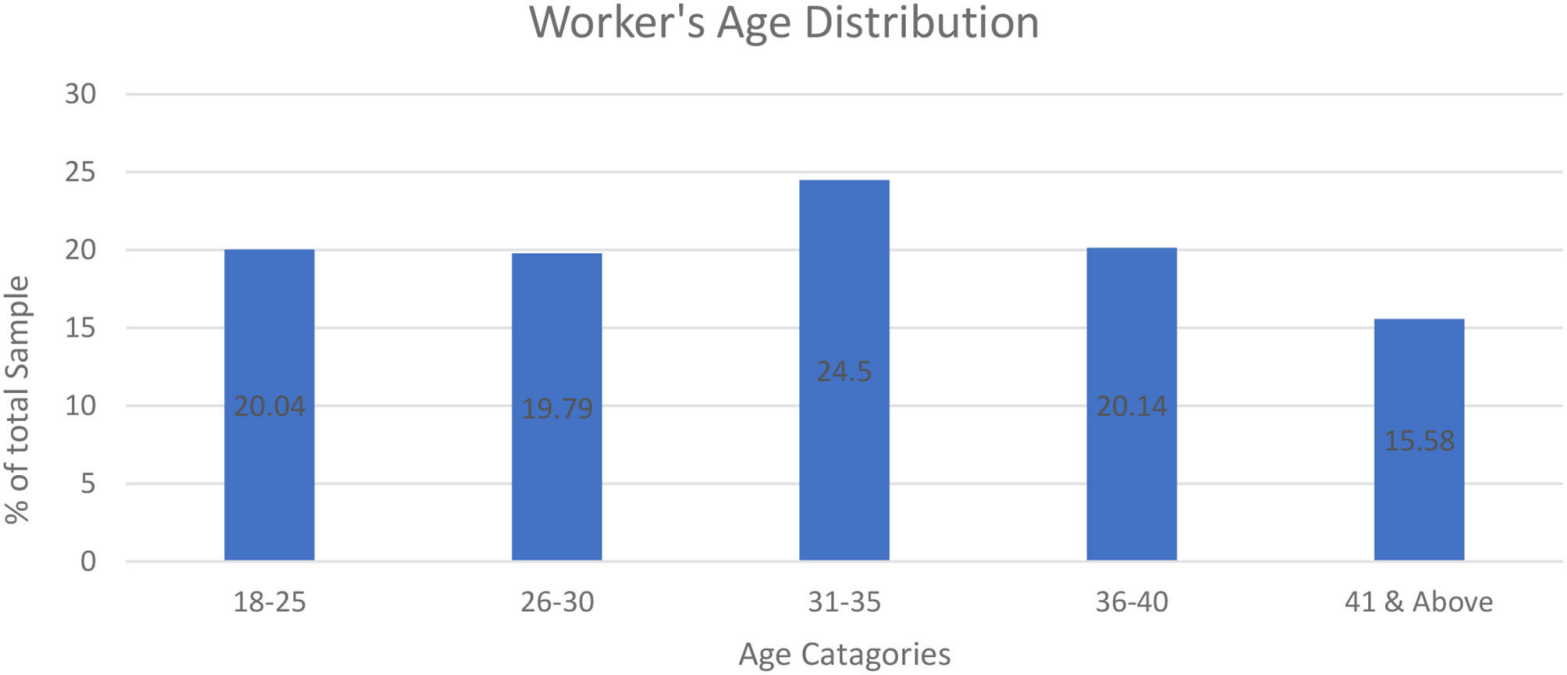
Workers' age distribution of Chinese SMEs.

The graphical analysis is conducted to reveal age distribution and COPD symptoms within the sample set and subsequently depicted in Figures 1, 2. Around 42 and 34% of workers have coughing during duty hours and breath shortness in working, respectively. It indicates that COPD is damaging the workers' health. As 70% of Japanese workers are employed in SMEs and have larger proportions of health problems as compared with workers in larger enterprises (Economic Census of Japan, 2012), age distribution illustrates that 40% of workers are young and below 30 years old.

Correlation analysis helps us to examine the associations among concerned variables of the study (see Table 1). Chronic obstructive pulmonary disease is positively associated with workers' health. There is a strongly consistent and most significant link between COPD and occupational exposures across and within industry groups, and they also found risk factors for COPD (35). On the other hand, the working condition and tools (WCT), smoking years (SY), wages (WG), and health safety training (HS) are negatively associated with workers' health. The aforementioned features can improve worker health by skipping smoking, better salaries, and safe training, respectively (13). A high occurrence of COPD among non-smoking females in Africa, the Middle East, Asia, and America are considered to be caused by the extensive usage of cooking fuels for biomass (13, 33). The occurrence of asthma was also higher among smoker's workers (34).

**Table 1:** Correlations matrix.

**Variables**	**WH**	**COPD**	**WCT**	**SY**	**WG**	**HS**
WH	1.000					
COPD	0.009	1.000				
WCT	−0.085	0.075	1.000			
SY	−0.295	−0.066	0.027	1.000		
WG	−0.085	0.075	1.000	0.027	1.000	
HS	−0.092	0.047	0.102	0.111	0.102	1.000

### Impact Analysis

Based on the maximum likelihood approach, the logistics regression is utilized to tackle the binary nature of the target variable, i.e., workers' health. It is also used to compute the impact of COPD on workers' health of Chinese SMEs. Table 2 represents the marginal estimates of logistics evaluation. The marginal estimates infer that a rise in COPD concerning wages will uplift the workers' health problems due to COPD affecting workers' health. This finding is similar to the study of Omland et al. (35). It also found a strong, consistent, and most significant link between COPD and occupational exposures across and within industry groups, and they also found risk factors for COPD, such as vapors, dust, gas, and fumes (35). The working condition and tools, smoking years, and health safety training have a statistically adverse effect on workers' health concerning wages. An increase in these features decreases the probability of COPD affects the workers' health. Owners of SMEs often manage manifold responsibilities, but limited resources and lack of formal training and experience could not focus on health workers (14).

**Table 2:** Logistics regression estimates (*N* = 1,008).

	**Marginal estimates**	**St.Dev**	**Z-stats**	***P* > |z|**
**LOGISTICS MODEL (TARGET VARIABLE** **=** **WH, WG** **=** **BASE VARIABLE)**				
COPD	0.0646^*^	0.0182	3.55	0.00
WCT	−0.0439^*^	0.0167	−2.63	0.00
SY	−0.2892^*^	0.0339	−8.51	0.00
HS	−0.0294^**^	0.0136	−2.16	0.031
**Diagnostics statistics**				
Logistic GOF	194.15^*^			
Pearson chi^2^	0.0004			
Link Test				
_hat	0.7986^*^	0.1336	5.98	0.00
_hatsq	−0.3613^**^	0.1655	−2.18	0.029
_cons	0.1373	0.0931	1.47	0.140
	**VIF**	**TOL** **=** **1/VIF**		
**VIF STATISTICS**				
COPD	1.03	0.966532		
WCT	1.01	0.986672		
SY	1.04	0.964581		
HS	1.03	0.967583		
Mean VIF	1.03			

* *1% and*

** *5% significant levels*.

The goodness of fit (GOF) of the estimated model is significant at 1% and infers that estimates are robust and reliable. The link test also affirms the validity of the model by inferring the correct functional form. It concludes that there is no problem with omitted variables and functional form as this study has been conducted using primary data. Therefore, multicollinearity must be addressed to avoid unstable estimates. To tackle this issue, VIF and TOL have been calculated to count the multicollinearity level. If the VIF value equals 10 or more and the tolerance value equals 0.1 or more, it may be essential to be advance examined (38). The estimated values of mean VIF and TOL are fine and fulfilled the acceptable criteria (see Table 2).

### Robustness Check: Accuracy and Validity

**Figure 3:**
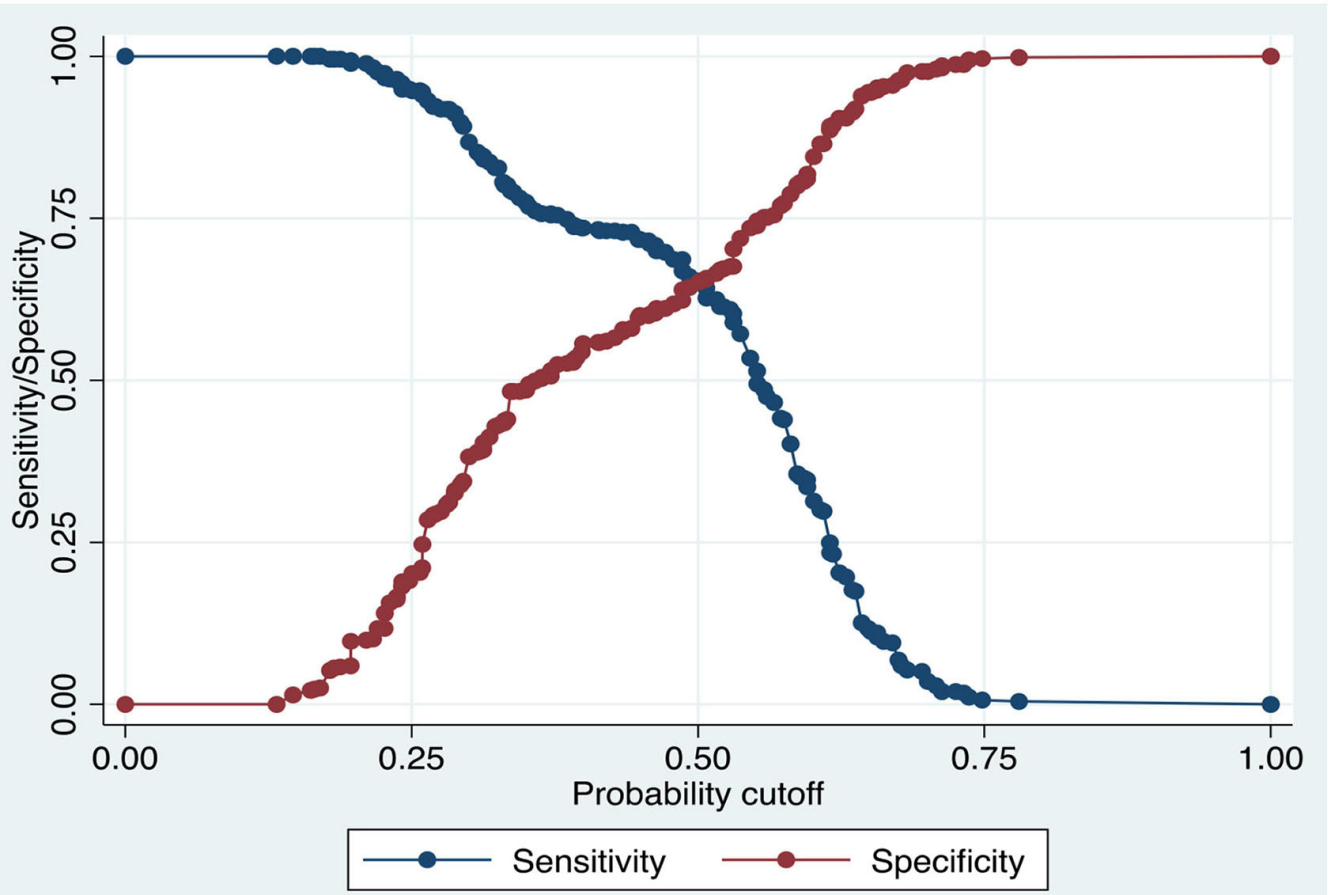
Model validity and accuracy analysis.

**Figure 4:**
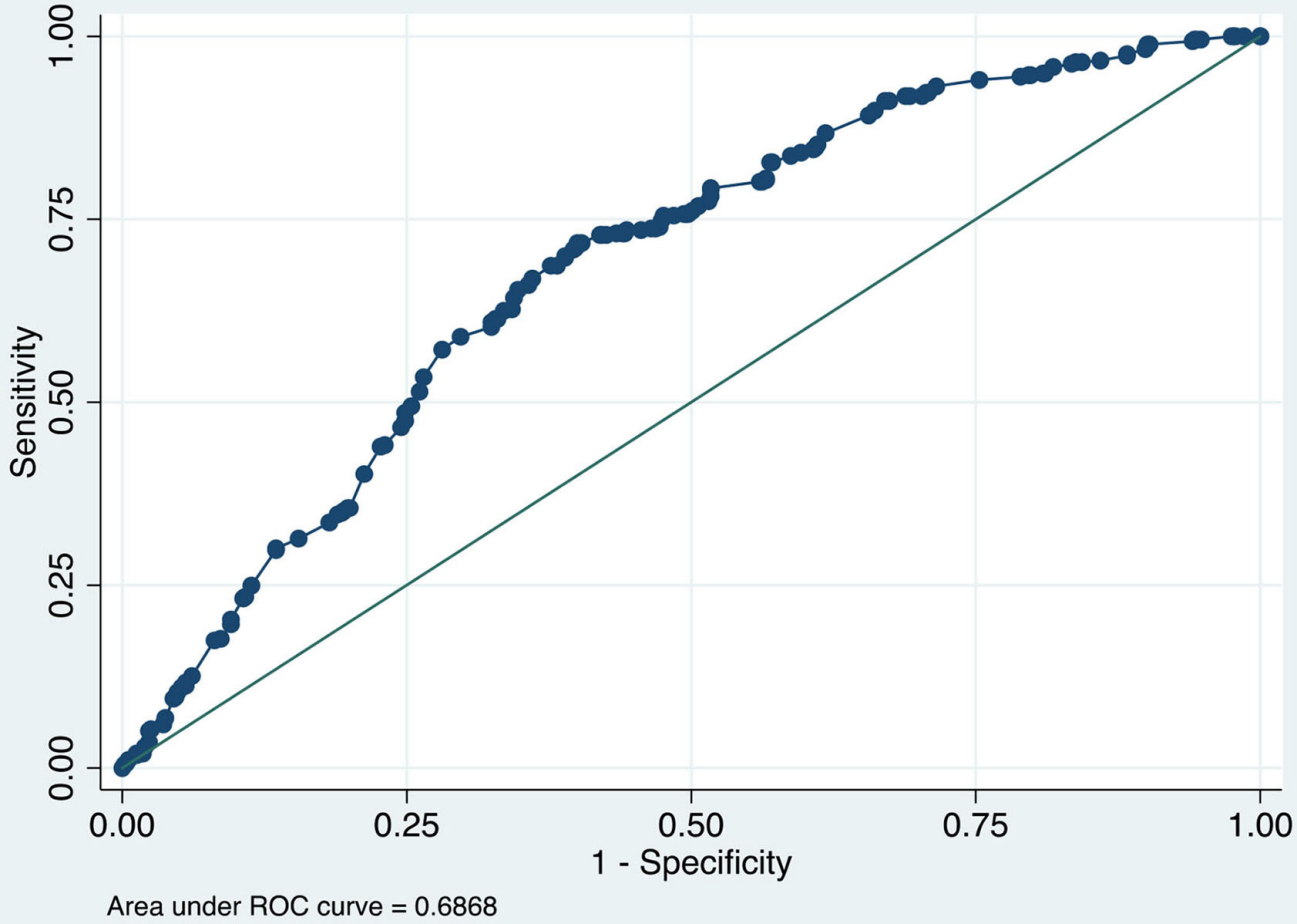
ROC analysis.

To affirm the estimates' validity, model accuracy and receiver operating characteristics curve (ROC) analysis have been conducted and reported in Table 3 and Figures 3, 4. Model accuracy has two components; sensitivity and specificity to spotlight the accuracy of the estimated model, which is true positive [Pr (+| D)] and [Pr (–|~D)] negative, respectively. Simply, it can be stated as the proportion of observed positives and negative to be predicted positives and negative, respectively (38, 39). It is a kind of trade-off between sensitivity and specificity. The estimated logistic model is 65.28% correctly specified.

**Table 3:** Sensitivity and specificity analysis.

	**True**				
**Classification**	**D**	****~**D**	**Total**		
**Logistic model for WH**					
**+**	296	193	489		
**–**	157	362	519		
Total	453	555	1,008		
**Cross-validation analysis**					
Sensitivity	Pr (+|D)	65.34%	Positive predictive value	Pr (+|D)	60.53%
Specificity	Pr (–|~D)	65.23%	Negative predictive value	Pr (–|~D)	69.75%
False – rate for true D	Pr (–|D)	34.77%			
False + rate for true ~D	Pr (+|~D)	34.66%			
False + rate for classified	Pr (~D|+)	39.47%			
False – rate for classified	Pr (D|–)	30.25%			
Correctly classified		65.28%			

The ROC or area under the curve (AUROC) is used to measure the validity of the estimated model. Figure 4 illustrates the AUROC and infers 0.6868 values which fall among the acceptable value, i.e., 0.5–1.0 (39). It concludes that AUROC predicted 68.68 percent correctly values.

## Conclusion

COPD has a high economic burden for each economy. Collaborators (1) reported that COPD becomes the third leading source of death and the sixth source of disability worldwide at the end of 2030. The economies, especially the emerging ones, are primarily focusing on every indicator to uplift their businesses. Along with the other aspects, it is also required to assess the impact of COPD on workers' health conditions in SMEs. With this aim, we are presenting a detailed analysis to reveal valuable insights regarding the COPD workers' health nexus. This study also attempts to explore the determinants of COPD in SMEs. Unlike prevalent precedencies, Probit, logit models, and state-of-the-art econometric approaches are used for region-wise predictive and non-linear analysis.

The marginal estimates infer that a rise in COPD concerning wages will uplift the worker health problems due to COPD affecting worker health. Whereas, the working condition and tools, smoking years, and health safety training have a statistically adverse effect on workers' health concerning wages. However, SMEs have limited resources, are more financially pressured, and have few work-related safety and health programs than their large enterprises.

## Policy Implications

The outcomes of this study can be helpful to devise policies related to labor laws and social welfare. The Chinese SME workers' health can be improved by providing better working conditions and tools and health safety training. Incremental taxation on smoking can also be helpful to improve workers' health. The results of this study will help to develop labor laws and social welfare policies. Through better working conditions and instruments and health protection training, Chinese small- and medium-sized businesses' health can be improved. Increased smoking taxes will also help improve the health of employees.

## Data Availability Statement

The original contributions presented in the study are included in the article/supplementary material, further inquiries can be directed to the corresponding author/s.

## Author Contributions

L-PY: conceptualization, software, data curation, and writing (original draft preparation). RT: methodology, visualization, investigation, and writing (reviewing and editing). All authors contributed to the article and approved the submitted version.

## Conflict of Interest

The authors declare that the research was conducted in the absence of any commercial or financial relationships that could be construed as a potential conflict of interest.
